# Determination of drug-related problems in the hematology service: a prospective interventional study

**DOI:** 10.1186/s12885-024-12291-w

**Published:** 2024-05-02

**Authors:** Aslınur Albayrak, Demircan Özbalcı

**Affiliations:** 1 Department of Clinical Pharmacy, Faculty of Pharmacy, Suleyman Demirel University, Isparta, Türkiye; 2 Department of Hematology, Faculty of Medicine, Suleyman Demirel University, Isparta, Türkiye

**Keywords:** Clinical pharmacist, Drug related problem, Hematologic malignancy

## Abstract

**Background:**

Patients with hematological malignancies often require multidrug therapy using a variety of antineoplastic agents and supportive care medications. This increases the risk of drug-related problems (DRPs). Determining DRPs in patients hospitalized in hematology services is important for patients to achieve their drug treatment goals and prevent adverse effects. This study aims to identify DRPs by the clinical pharmacist in the multidisciplinary team in patients hospitalized in the hematology service of a university hospital in Turkey.

**Methods:**

This study was conducted prospectively between December 2022 and May 2023 in the hematology service of Suleyman Demirel University Research and Application Hospital in Isparta, Turkey. DRPs were determined using the Pharmaceutical Care Network Europe (PCNE) 9.1 Turkish version.

**Results:**

This study included 140 patients. Older age, longer hospital stay, presence of acute lymphoblastic leukemia, presence of comorbidities, higher number of medications used, and polypharmacy rate were statistically significantly higher in the DRP group than in the non-DRP group (*p* < 0.05). According to multivariate logistic regression analysis, the probability of DRP in patients with polypharmacy was statistically significant 7.921 times (95% CI: 3.033–20.689) higher than in patients without polypharmacy (*p* < 0.001).Every 5-day increase in the length of hospital stay increased the likelihood of DRP at a statistically significant level (OR = 1.476, 95% CI: 1.125–1.938 *p* = 0.005). In this study, at least one DRP was detected in 69 (49.3%) patients and the total number of DRPs was 152. Possible or actual adverse drug events (96.7%) were the most common DRPs. The most important cause of DRPs was drug choice (94.7%), and the highest frequency within its subcategories was the combination of inappropriate drugs (93.4%).

**Conclusions:**

This study shows the importance of including a clinical pharmacist in a multidisciplinary team in identifying and preventing DRPs in the hematology service.

## Background

Hematological malignancies include a variety of diseases such as Hodgkin lymphoma, non-Hodgkin lymphoma, leukemias, and multiple myeloma [1]. New treatment strategies were developed for all these diseases and the survival time of patients was increased [2–4]. Hematological cancer patients require combination therapy using a variety of antineoplastic agents and supportive care medications [5]. Polypharmacy is the use of multiple medications and is common in this patient group [6]. Polypharmacy increases the risk of drug-related problems (DRPs) [7]. DRPs are defined as an event or situation involving medication that interferes with desired health outcomes. DRPs include inappropriate dosage and method of administration, drug-drug interactions, drug omissions and monitoring deficiencies, and adverse drug reactions [8, 9]. This may fail to achieve drug therapy goals or harm the patient [10]. It also causes prolonged hospital stay, readmission, and increased mortality [11–13].

Within a multidisciplinary team, clinical pharmacists can detect and prevent DRPs early through comprehensive medication review [9, 14]. Clinical pharmacy services are pretty new in Turkey. Although there have been postgraduate programs (master’s degree, doctorate) related to clinical pharmacy for years, there has been a clinical pharmacy specialty program since 2018 [15]. Only graduates of the clinical specialty program can work in public hospitals [16]. Therefore, the number of clinical pharmacists actively working in hospitals is relatively low.

The contributions of clinical pharmacists in identifying and preventing DRPs have been demonstrated in many clinical departments [14, 17–20]. However, studies on determining DRPs in patients with hematological malignancy are limited [5, 9, 21–23]. In a study conducted in an onco-hematology and bone marrow transplant unit in Brazil [23], the frequency of DRPs was found to be 135 (9%). 135 interventions were performed by the pharmacist and 90% were accepted. In a study conducted in France [9], 552 (12.6%) DRPs were found. Medication problems were mostly related to anti-infective agents, and oncologists’ acceptance of interventions was found to be high (96%). In a study conducted in Korea [5], a total of 1187 DRPs were identified in 438 (23.9%) of 1836 hospitalized patients with hematological malignancy. Pharmacists’ intervention was accepted by 88.3%. In a study examining the clinical and economic impact of pharmacist interventions in an outpatient hematology-oncology department in France [24], a total of 1970 pharmacist interventions were performed, corresponding to an average of 3.5 pharmacist interventions/patient, and the total cost savings was €175,563. The clinical pharmacist’s cost-benefit ratio was found to be €3.7 for every €1 invested.

As far as it is known, no study shows that DRPs are determined by the clinician in the hematology service in Turkey. Therefore, this study aims to determine drug-related problems by a clinical pharmacist within the multidisciplinary team in patients with a diagnosis of hematological malignancy hospitalized in the hematology services of a university hospital in Turkey.

## Methods

### Study design

This study was conducted prospectively between December 2022 and May 2023 in the hematology service of Suleyman Demirel University Research and Application Hospital in Isparta, Turkey.

All patients over the age of 18 who were hospitalized in the hematology service for more than 24 h were included in the study. Only the first hospitalization of each patient was evaluated. Informed consent was obtained from all participants before they participated in the study. Ethics Committee approval was obtained from Suleyman Demirel University Faculty of Medicine Clinical Research Ethics Committee (Approval No:274, Date:28.09.2022).

### Setting

The service where the research was conducted had 15 beds and two physicians and assistant physicians were working. There was no stem cell transplant unit in the hospital. Isparta was a small city with a population of 449,777 [25]. The hospital and patient population where the study was conducted were smaller than the hospitals in Turkey’s metropolitan cities.

### Sample size

The sample size was calculated based on the approximate number of patients admitted to the hematology service during the previous 6 months. With the Raosoft sample size calculator, the sample size was found to be minimum 123 with a population size of 180, 5% margin of error, 95% confidence interval and 50% distribution rate [26].

### Data collection

The clinical pharmacist in the study was an academic, did not routinely work in this hospital, and was present at the hospital for this study. The clinical pharmacist performed comprehensive medication reviews of patients and provided interventions. The patients’ socio-demographic characteristics, history, diagnosis, comorbidities, medications used, laboratory test results, and interventions were recorded in the data collection form by the clinical pharmacist. The patients’ data were obtained from the hospital database, patient files, and patients. In general, interventions were made through verbal communication. UpToDate® and Sanford Guide to Antimicrobial Therapy Mobile® software were used for the interventions [27, 28]. The Lexicomp Drug Interactions® tool, accessed via UpToDate®, was used to identify drug-drug interactions [29]. According to Lexicomp Drug Interactions®, drug interactions consist of five categories. A -no known interaction, B- no action required, C -monitor therapy, D- consider changing therapy, X- avoid combination. The presence of at least one of the risk levels C, D, and X was defined as a potential drug-drug interactions because it was clinically significant [30–32]. Polypharmacy was defined as the use of 5 or more medications [33, 34].

DRPs were determined using the Pharmaceutical Care Network Europe (PCNE) 9.1 Turkish version. PCNE 9.1 has 3 primary fields for problems, 9 primary fields for causes, 5 primary fields for planned interventions, 3 primary fields for acceptance level (of interventions), and 4 primary fields for status of the problem. Problems include treatment effectiveness and safety, while reasons include drug selection, drug form dose selection, and treatment duration [35].

### Statistical analysis

Statistical analysis was performed using SPSS 20. Continuous variables were expressed as median-interquartile range, and categorical variables were expressed as percentage and frequency. The normality of the data was analysed with the Kolmogorov-Smirnov test. The Mann-Whitney U test was used to compare continuous independent variables, and the Chi-Square test was used for categorical variables. The Pearson Chi-Square (> 25), the Continuity Correction (5–25), and the Fisher’s Exact test (< 5) were used according to the number of cases. Multiple logistic regression analysis was performed to determine the best predictor(s) which effect on the presence of DRP. Any variable whose univariable test had a p value < 0.10 was accepted as a candidate for the multivariable model along with all variables of known clinical importance. Odds ratios, 95% confidence intervals and Wald statistics for each independent variable were also calculated. A p-value smaller than 0.05 was considered statistically significant.

## Results

This study included 140 patients. Almost half (55%) of the patients were male and the median age was 65 (55–74) years. The median length of hospital stay was 8 (5–14) days. The median number of medications used by the patients was 6 (4–7). Polypharmacy was present in 67% of the patients. Older age, longer hospital stay, presence of acute lymphoblastic leukemia, presence of comorbidities, higher number of medications used, and polypharmacy rate were statistically significantly higher in the DRP group than in the non-DRP group (*p* < 0.05). Table 1 shows the socio-demographic and clinical characteristics of the patients.

**Table 1:** Socio-demographic and clinical characteristics of the patients (*n* = 140)

	No DRP *n* (%)	Presence of DRP *n* (%)	*p*	Total *n* (%)
Gender				
Male	41 (56.2)	37 (55.2)	0.911	78 (55.7)
Female	32 (43.8)	30 (44.8)		62 (44.3)
Age, years (median, IQR)	62 (49.5–71)	68 (59–75)	**0.014**	65 (55–74)
Length of hospital stay (median, IQR)	7 (4–11)	11 (7–20)	**< 0.001**	8 (5–14)
Diseases				
Acute lymphoblastic leukemia	1 (1.4)	7 (10.4)	**0.028**	8 (5.7)
Acute myeloid leukemia	11 (15.1)	11 (16.4)	1	22 (15.7)
Chronic myeloid leukemia	2 (2.7)	**-**	0.497	2 (1.4)
Chronic lymphoblastic leukemia	8 (11)	5 (7.5)	0.567	13 (9.3)
Myelodysplastic syndrome	2 (2.7)	3 (4.5)	0.67	5 (3.6)
Non-Hodgkin’s lymphoma	10 (13.7)	12 (17.9)	0.652	22 (15.7)
Hodgkin’s lymphoma	4 (5.5)	1 (1.5)	0.368	5 (3.6)
Multiple myeloma	14 (53.8)	12 (46.2)	1	26 (18.6)
Aplastic anemia	1 (1.4)	1 (1.5)	1	2 (1.4)
Autoimmune hemolytic anemia	1 (1.4)	3 (4.5)	0.349	4 (2.9)
Iron deficiency anemia	4 (5.5)	1 (1.5)	0.368	5 (3.6)
Immune thrombocytopenic purpura	4 (5.5)	3 (4.5)	1	7 (5)
Others	11 (15.1)	8 (11.9)	0.770	19 (13.6)
Comorbidities	36 (49.3)	45 (67.2)	**0.033**	81 (57.9)
Cardiovascular	27 (37)	33 (49.3)	0.143	60 (42.9)
Diabetes mellitus	11 (15.1)	16 (23.9)	0.269	27 (19.3)
Thyroid	3 (4.1)	3 (4.5)	1	6 (4.3)
Neuropsychiatric disease	2 (2.7)	4 (6)	0.426	6 (4.3)
COPD/Asthma	4 (5.5)	7 (10.4)	0.352	11 (7.9)
Chronic liver disease	**-**	2 (3)	0.227	2 (1.4)
Chronic kidney disease	**-**	3 (4.5)	0.107	3 (2.1)
Others	3 (4.1)	-	0.246	3 (2.1)
Number of drugs (median, IQR)	4 (3–6)	7 (6–9)	**< 0.001**	6 (4–7)
Polypharmacy	34 (46.6)	60 (89.6)	**< 0.001**	94 (67.1)

COPD: Chronic obstructive pulmonary disease, n = number of patients, IQR: Interquartile range

At least one DRP was detected in 69 (49.3%) patients and the total number of DRPs was 152. Possible or actual adverse drug events (96.7%) were the most common DRPs. The most important cause of DRPs were drug choice (94.7%), and the highest frequency within its subcategories was the combination of inappropriate drugs (93.4%). Potential drug-drug interactions were detected in at least one C risk in 43 (30.7%) patients, at least one D risk in 11 (7.9%) patients, and at least one X risk in 6 patients (4.3%).

The clinical pharmacist performed 104 (68.4%) interventions on the prescriber, of which 100 (96.15%) were accepted and fully implemented. All 120 DRPs (78.9%) were resolved, and 28 DRPs (18.4%) were not possible or necessary to be resolved. Table 2 shows the classification of DRPs. Table 3 shows some examples of interventions performed by the clinical pharmacist. Anticancer drugs such as venetoclax, lenalidomide, and dasatinib were examples of potential drug-drug interactions. Table 4 shows the adverse effects that occurred. Drug-related nephrotoxicity was the most common adverse effect. Table 5 shows the results of the multivariate logistic regression analysis: factors most predictive of the presence of DRP. Polypharmacy and length of hospitalization were the most determinant factors in differentiating the groups with and without DRP, respectively. After adjustment for other factors, the likelihood of the presence of DRP was statistically significantly 7.921 folds (95% CI: 3.033–20.689) higher in patients with polypharmacy compared to patients without polypharmacy (*p* < 0.001). On the other hand, each 5-day increase in the duration of hospitalization continued to increase the likelihood of the presence of DRP by a statistically significant (OR = 1.476, 95% CI: 1.125–1.938 *p* = 0.005).

**Table 2:** Classification of drug-related problems in the patient population

Domains	*N* (%)
Drug related problems detected	152
Frequency of DRPs in patients	69 (49.3)
Potential or manifest problems	
P1. Treatment effectiveness	
P1.2 Effect of drug treatment not optimal	3 (1.97)
P2. Treatment safety	
P2.1 Adverse drug event (possibly) occurring	147 (96.7)
P3.1 Unnecessary drug-treatment	2 (1.31)
Potential or manifest causes	
C1. Drug selection	144 (94.7)
C1.3 Inappropriate combination of drugs, or drugs and herbal medications, or drugs and dietary supplements	142 (93.4)
C1.4 Inappropriate duplication of therapeutic group or active ingredient	1 (0.66)
C1.6 Too many different drugs/active ingredients prescribed for indication	1 (0.7)
C3. Dose selection	8 (5.26)
C3.1 Drug dose too low	1 (0.66)
C3.2 Drug dose of a single active ingredient too high	4 (2.63)
C3.4 Dosage regimen too frequent	2 (1.31)
C3.5 Dose timing instructions wrong, unclear or missing	1 (0.66)
C6. Drug use process	
C6.1 Inappropriate timing of administration or dosing intervals by a health professional	1 (0.66)
The planned interventions	
I0.1 No Intervention	12 (7.89)
I1.At prescriber level	
I1.1 Prescriber informed only	35 (23)
I1.3 Intervention proposed to prescriber	104 (68.4)
I1.4 Intervention discussed with prescriber	1 (0.65)
I3. At drug level	
I3.1 Drug changed to	7 (4.6)
I3.2 Dosage changed to	66 (43)
I3.4 Instructions for use changed to	10 (6.57)
I3.5 Drug paused or stopped	17 (11.16)
I3.6 Drug started	7 (3.94)
Acceptance of the Intervention proposals	
A.1 Intervention accepted	
A1.1 Intervention accepted and fully implemented	100 (96.15)
A.2 Intervention not accepted	
A2.1 Intervention not accepted: not feasible	2 (1.92)
A2.2 Intervention not accepted: no agreement	2 (1.92)
Status of the DRP	
O 1 Solved	
O1.1 Problem totally solved	120 (78.9)
O3 Not solved	
O3.2 Problem not solved, lack of cooperation of prescriber	4 (2.63)
O3.4 No need or possibility to solve problem	28 (18.4)

**Table 3:** Examples of interventions performed by a clinical pharmacist

DRPs category	Example interventions
**Drug selection**	
Inappropriate combination of drugs, or drugs	
Venetoclax- verapamil trandolapril	Since verapamil-trandolapril is a CYP3A4 inhibitor, it may increase the serum concentration of Venetoclax. It was recommended to reduce the venetoclax dose by 50%.
Dasatinib-pantoprazole	Since pantoprazole may reduce the concentration of dasatinib, an antacid agent (with a 2-hour time difference) was recommended.
Amikacin-vancomycin	Vancomycin may increase the risk of nephrotoxicity of amikacin. Patients were monitored for creatinine levels, and dose adjustment of amikacin was recommended according to the patient’s CrCl.
Bortezomib-diltiazem	Since diltiazem is a CYP3A4 inhibitor, it may increase bortezomib concentrations.The prescriber was informed and bortezomib was monitored for toxicity.
Pantoprazole-methotrexate	Pantoprazole may increase the concentration of methotrexate. The prescriber was informed. Methotrexate was monitored for toxicity.
Bortezomib-tamsulosin	The blood pressure lowering effect may be increased when both drugs are used together. The patient was monitored for hypotension.
Cytarabine- amphotericin B	Cytarabine may increase the nephrotoxic effect of amphotericin B. The patient was monitored for creatinine values and the prescriber was informed.
Vancomycin-piperacillin tazobactam	Piperacillin tazobactam may increase the nephrotoxic effect of vancomycin. Since the patient’s creatinine values were high, it was recommended to reduce the dose of piperacillin tazobactam.
Inappropriate duplication of therapeutic group or active ingredient	Tiotropium bromide-ipratropium bromide It was recommended to discontinue ipratropium bromide.
**Dose selection**	
Drug dose too low	Piperacillin tazobactam-Since the patient’s CrCl increased compared to the previous one, it was recommended to increase the piperacillin dose from 3.375 g in 6 h to 4.5 g in 6 h.
Drug dose of a single active ingredient too high	Tramadol-Since the patient had CrCL < 30, it was recommended not to exceed 200 mg daily.
Dosage regimen too frequent	Amicasin - Due to renal dysfunction, it was recommended to give the dose every 48 h.
Inappropriate timing of administration or dosing intervals by a health professional	Plasmapheresis- amlodipine-Since it is highly protein bound, the possibility of drug removal in plasmapheresis was found to be high. According to the literature, it was recommended that the drug be either given in high doses or given after plasmapheresis.

CrCl: Creatinine clearance, DRPs: drug related problems, g:gram, mg: milligram

**Table 4:** Examples of observed adverse drug events

Drugs	Adverse effect (*n*)
Amikacin	Nephrotoxicity (1)
Amphotericin B liposomal	Nephrotoxicity (4)
Vancomycin	Nephrotoxicity (3)
Furosemide	Nephrotoxicity (1)
Cisplatin	Nephrotoxicity (1)
Rituximab	Skin rash (1)
Venetoclax	Hyperuricemia, hyperkalemia (1) Neutropenia (1)
Levofloxacin	Levofloxacin hypersensitivity-anaphylaxis (1)
Methotrexate	Increased liver enzymes (1)
Hydrochlorothiazide	Hypokalemia (1)
ABVD chemotherapy protocol	Anemia (1)
Vincristine	Neuropathy (1)

**Table 5:** Factors most predictive of the presence of DRP: results of multivariate logistic regression analysis

	OR	95% CI	Wald	*p*
Age	1.025	0.998–1.053	3.290	0.070
Length of hospital stay ^*^	1.476	1.125–1.938	7.878	**0.005**
Comorbidity	2.036	0.846–4.899	2.519	0.112
Polypharmacy	7.921	3.033–20.689	17.850	**< 0.001**

OR: Odds ratio, CI: Confidence interval. * The effect of each 5-day increase in the duration of hospitalization

## Discussion

In our study, 152 DRPs were identified and 120 DRPs were totally solved. This reveals the importance of involving the clinical pharmacist in a multidisciplinary team. The most common DRPs in our study were possible or actual adverse drug events. Since the patient population was generally elderly and cancer patients, they were exposed to polypharmacy and drug-drug interactions. Additionally, this was not surprising since the risk of exposure to possible or actual adverse drug events was high due to the anticancer medications they use [36, 37]. Adverse drug events varied across studies. While this rate was 28.6% in the study conducted by Kim et al. [5] in the hematology service, it was 78.6% in the study conducted by Umar et al. [14] in the oncology service. Since Kim et al.‘s study [5] was retrospective, the rate of possible or actual adverse effects may have been found to be low. Additionally, although both studies used the PCNE classification system, it was not mentioned in Kim et al.‘s study which drug-drug interaction tool was used and which risk ratio for drug-drug interaction was considered clinically significant.

​In our study, most of the causes of DRPs were related to drug selection and their subgroup, inappropriate combination of drugs. Drug-drug interaction rates in the studies were 14.3%, 7.4%, 13.6%, and 73.2%, respectively [5, 9, 14, 23]. Differences in this rate may be due to polypharmacy rates, differences in healthcare services, and different drug-drug interaction software [38, 39]. Most of the potential drug-drug interactions in our study were at risk C (monitor therapy). Therefore, in some drug-drug interactions that required monitoring, only the physician was informed, and in others, intervention was recommended to the prescriber. Drug-drug interactions were mostly related to supportive medications. In our study, anticancer drugs such as venetoclax, lenalidomide, bortezomib, and dasatinib had potential drug-drug interactions. Venetoclax had potential drug-drug interactions with verapamil-trandolapril at increased risk of D. Verapamil-trandolapril is a CYP3A4 inhibitor [40], and concomitant use with venetoclax increases the concentration of venetoclax. It is recommended that the dose of venetoclax be reduced by 50% [29, 41–43]. Also, there was a potential drug-drug interaction at risk X (avoid combination) between dasatinib and pantoprazole. Concomitant use of these two agents decreases the concentration of dasatinib [44]. Bortezomib had potential drug-drug interactions at risk level C with antihypertensive drugs and drugs used in the treatment of benign prostatic hyperplasia, such as tamsulosin [29]. Bortezomib may have a blood pressure-lowering effect, so if used concomitantly with an antihypertensive drug or another drug that can lower blood pressure, the patient should be monitored for hypotension [45, 46]. In our study, there was also a potential drug-drug interaction between bortezomib and diltiazem at risk level C. Diltiazem, as a CYP3A4 inhibitor, may increase bortezomib concentration [40]. The bortezomib prescribing information emphasizes that in this case, it should be monitored for toxicity and dose reduction should be made if necessary [29, 47]. In our study, there was a potential drug-drug interaction between lenalidomide and dexamethasone. When lenalidomide and dexamethasone are used together, venous thromboembolism prophylaxis should be considered, as the thrombogenic activity of lenalidomide may increase [29, 48, 49]. Additionally, potential drug-drug interactions with antiemetics and opioid-derived analgesics were frequently observed in our study. Identifying, monitoring, and intervening when necessary, drug-drug interactions are very important in cancer patients, and clinical pharmacists have important roles in this regard [50, 51].

Dose selection was the second important DRP in our study. Renal dosage adjustment of drugs is significant, especially in patients who develop acute kidney injury [52]. Even if the drugs are started at the correct dose, the dose of the drugs should be monitored and adjusted when necessary in case of liver and renal dysfunction [52, 53]. In our study, antimicrobials were among the drugs that required dosage adjustment according to renal function. This was due to the fact that although infectious disease physicians started antimicrobials at the correct dose, these doses were sometimes not followed up later.

Drug-induced nephrotoxicity was a common adverse event in our study, similar to other studies [17, 54]. Also, venetoclax-related hyperuricemia, hyperkalemia and neutropenia were observed in some patients. In a study investigating the incidence of venetoclax-related toxicity risk in British Columbia, hyperkalemia and hyperphosphatemia were observed in 9 patients (27%), and hyperuricemia was observed in 7 patients (21%) [55]. In their study by Koehler et al., venetoclax-related hyperkalemia (31%) and hyperuricemia (5%) were observed [56]. In our study, one acute lymphoblastic leukemia patient had vincristine-induced neuropathy. Vincristine-induced neuropathy is a common side effect and its incidence is between 30 and 40% [57].

The clinical pharmacist’s acceptance rate of the interventions was good. In general, interventions regarding renal and hepatic dosing were accepted. The clinical pharmacist did not intervene in some cases that required monitoring (for example, category C drug interactions) and only informed the physician. These were evaluated as not possible or necessary to resolve the problem.

One of the strengths of the study is that the acceptability of the interventions was higher than other studies [5, 18, 23, 58]. Additionally, our study was the first study in Turkey to reveal DRPs in detail in this vulnerable patient population in the hematology service. One of the limitations of our study is that it was conducted in a single center and with a small number of patients. In addition, the clinical pharmacist in the study was an academician and did not work full-time in the hospital, but worked at certain times of the day. This may have caused some DRPs not to be determined.

## Conclusion

According to our study, a high frequency of DRPs and possible or actual adverse drug events were detected in patients. Older age, longer hospital stay, presence of acute lymphoblastic leukemia, presence of comorbidities, higher number of medications used, and polypharmacy rate were statistically significantly higher in the DRP group than in the non-DRP group According to the results of multiple logistic regression analysis, polypharmacy and length of hospital stay were the most determining factors in distinguishing between groups with and without DRP. The most common DRP was related to possible or actual adverse drug events. The most common cause of DRPs was drug selection and its subgroup, inappropriate combination of drugs. Also, our study shows the importance of including a clinical pharmacist in a multidisciplinary team in identifying and preventing DRPs in the hematology service.

## Data Availability

The datasets used and/or analysed during the current study available from the corresponding author on reasonable request.
